# Raised levels of latent collagenase activating angiogenesis factor (ESAF) are present in actively growing human intracranial tumours.

**DOI:** 10.1038/bjc.1991.262

**Published:** 1991-07

**Authors:** C. M. Taylor, J. B. Weiss, R. H. Lye

**Affiliations:** Department of Rheumatology, University of Manchester, UK.

## Abstract

**Images:**


Br. J. Cancer (1991), 64, 164-168 © Macmillan Press Ltd., 1991

### Raised levels of latent collagenase activating angiogenesis factor (ESAF) are present in actively growing human intracranial tumours

C.M. Taylor[1], J.B. Weiss[1] & R.H. Lye[2]

*Departments of [1]Rheumatology and [2]Neurosurgery, University of Manchester, Manchester M13 9PT, UK.*

**Summary** Endothelial cell stimulating angiogenesis factor (ESAF) is a potent, low molecular mass mitogen, specific for endothelial cells. In common with various protein growth factors, it displays angiogenic activity in a variety of biological test systems. However, it differs from these other factors by virtue of its low molecular mass and its ability to activate latent matrix metalloproteinases in a dose dependent manner. This activity has been used to quantify the factor in both normal and diseased brain tissue. The concentration of ESAF determined in biopsies from different types of intracranial tumours varied: in some tumour types the level was close to that of control samples whereas in others it rose to levels comparable to those encountered in the pineal gland, the richest source of ESAF in mature mammals. Tumours considered to be benign contained significantly less ESAF than those neoplasms classified as being malignant ($P = 0.025$). There was also a correlation between the mitotic activity of tumour samples, as determined by conventional H & E histochemical staining and the ESAF concentration present. These findings agree with previous studies in which elevated ESAF levels have been found in tissue where proliferation of vascular elements has been observed.

The continual growth of a tumour beyond a critical size relies on the development of new blood vessels (neovascularisation) to maintain an adequate supply of nutrients for the tumour cells (Folkman, 1984). It is known that tumours produce several substances capable of inducing the proliferation and migration of host capillary endothelial cells. These substances include fibroblast growth factors (Shing *et al.*, 1985; Klagsbrun *et al.*, 1986), which are non-specific cell mitogens widely distributed in neural and other tissues. Recently it has been shown that the stimulation of microvessel cell proliferation by these factors is enhanced if endothelial cell stimulating angiogenesis factor (ESAF) is present (Odedra & Weiss, 1987).

The molecular mass of ESAF is approximately 400 and its angiogenic activity has been demonstrated in several test systems (Weiss *et al.*, 1987; Elstow *et al.*, 1985). Unlike protein growth factors, ESAF is a mitogen specifically for microvessel cells (Schor *et al.*, 1980). It enhances the chemotaxis and chemokinensis of microvessel, but not aortic endothelial cells and has no effect on fibroblasts (Odedra and Weiss in preparation). The synergism between fibroblast growth factors and ESAF suggests the presence of the latter may be vital for the *in vivo* stimulation of capillary growth.

It has been shown that ESAF activates latent collagenase (Weiss *et al.*, 1983) by reversing the inhibition of this enzyme by tissue inhibitor of metalloproteinases (McLaughlin *et al.*, 1991). These findings suggest that ESAF has an important role in the connective tissue destruction associated with blood vessel penetration. It is also probable that ESAF is implicated in the connective tissue breakdown associated with the expansion of neoplasms.

### Materials and methods

### *Assessment of tumours*

At operation, as large a biopsy as possible was taken from each tumour and stored at $-20°C$ until processing for the extraction of ESAF. Cautery was avoided when the biopsy was obtained. As far as could be ascertained, the biopsy samples taken were typical of the tumour under consideration.

Correspondence: J.B. Weiss, Wolfson Angiogenesis Unit, Department of Rheumatology, Hope Hospital, Salford M6 8HD, UK.
Received 9 November 1990; and in revised form 19 February 1991.

Tumour classification was based upon histological examination of biopsy specimens taken adjacent to the area where the samples for ESAF determination had been obtained. Tissue staining techniques used the standard haemotoxylin and eosin method and samples were classifed as 'actively growing' according to the number of mitotic figures seen, areas of necrosis and vascularity (Vafardis *et al.*, 1987).

### *Tumour and control brain samples*

Biopsy samples of tumour tissue was obtained from 20 patients with benign or malignant intracranial tumours (Tables I and II). Control brain tissue was obtained at autopsy from a variety of non-tumour patients or from patients with head injuries when damaged brain was resected (Table III). Samples were frozen at $-20°C$ prior to extraction.

### *Extraction of ESAF*

Individual samples were homogenised at 4°C in 50 mM $NH_4HCO_3$ buffer (pH 7.9) containing 2 M Mg $Cl_2$. After centrifugation at 20000 *g* (1 h, 4°C) the supernatant was assayed for protein (Lowry *et al.*, 1951) prior to ultrafiltration on a YM5 filter membrane (5000 Mr exclusion limit) (Amicon, Stonehouse, Glou., UK) with five volumes of bicarbonate buffer. The ultrafiltrate was reduced by rotary evaporation to a volume of 5 ml and applied to an octodecyl silica column (Analytichem, Harbor City, California, USA). Bound low molecular mass material was eluted with methanol.

Assay of angiogenic material for its ability to activate latent collagenase was performed according to the method of Weiss *et al.* (1983). Results were expressed as $\mu g$ collagen degraded $h^{-1} mg^{-1}$ protein in the supernatant.

The active material eluting from the octodecyl silica column passed readily through a membrane with an exclusion limit of 3000 Mr. The apparent molecular mass was established by gel filtration on a Bio-Gel P-2 (Weiss *et al.*, 1979). Angiogenic activity was assessed using the chick yolk sac membrane test (Taylor & Weiss, 1984).

### *Latent collagenase activation assay*

$^3H$-labelled rat tail tendon collagen was prepared by a modification of the method of Gisslow and McBride (1975). Latent collagenase was prepared from the culture fluid of human skin fibroblasts stimulated with cytochalasin B. After concentration × 100 the fluid was applied to an ACA col-

**Table I** Benign intracranial tumours

| Patient no. | Sex | Age | Tumour site | Tumour type | Histological features | Signs of active growth[a] | Drug treatment | Recurrence[b] | Mean ESAF content μg collagen degraded $h^{-1}$ $mg^{-1}$ protein |
|---|---|---|---|---|---|---|---|---|---|
| 1 | M | 71 | Frontal convexity | Meningioma | Psammomatous | + | Steroids + folic acid | No | 1.42 |
| 2 | F | 62 | Olfractory groove | Meningioma | Meningothelial | – | Steroids | No | 0.235 |
| 3 | M | 65 | Frontal parasagittal | Meningioma | Meningothelial | + + | None | No | 4.80 |
| 4 | M | 60 | Frontal convexity | Meningioma | Haemangioblastoma | – | Steroids | No | 1.43 |
| 5 | F | 60 | Cerebello-pontine angle | Meningioma | Fibrous | + | Nifedipine | No | 1.385 |
| 6 | F | 58 | Pituitary fossa | Pituitary | Prolactinoma | – | Steroids | No | 1.68 |
| 7 | M | 63 | Pituitary fossa | Pituitary | GH-secreting adenoma | – | Steroids | No | 0.40 |
| 8 | M | 55 | Pituitary fossa | Pituitary | FSH-secreting adenoma | – | Steroids | No | 3.66 |
| 9 | M | 55 | Cerebello-pontine angle | Acoustic nerve tumour | Schwannoma Antoni A & B | – | Steroids | No | 0.38 |
| 10 | M | 37 | Cerebello-pontine angle | Acoustic nerve tumour | Schwannoma Antoni A & B | – | Steroids | No | 0.79 |
| 11 | M | 51 | Cerebello-pontine angle | Acoustic nerve tumour | Schwannoma Antoni A & B | – | None | No | 1.18 |
| 12 | F | 27 | Cerebello-pontine angle | Acoustic nerve tumour | Schwannoma Antoni A & B | – | None | No | 0.15 |

[a]Any one (+) or more (+ +) of: nuclear pleomorphism; cellular immaturity; tumour angiogenesis; tumour necrosis. [b]To some extent recurrence depends upon lengths of clinical follow up or survival.

**Table II** Malignant intracranial tumours

| Patient no. | Sex | Age | Tumour site | Tumour type | Histological[c] features | Signs of active growth[a] | Drug treatment | Recurrence[b] | Mean ESAF content μg collagen degraded $h^{-1}$ $mg^{-1}$ protein |
|---|---|---|---|---|---|---|---|---|---|
| 13 | M | 45 | Occipital lobe | Glioma | Astrocytoma Grade III | + | Steroids | Died | 1.40 |
| 14 | M | 57 | Temporal lobe | Glioma | Astrocytoma Grade IV | + + | Steroids | Yes | 1.94 |
| 15 | M | 66 | Frontal lobe | Glioma | Astrocytoma Grade III–IV | + + | Steroids | Died | 4.05 |
| 16 | F | 65 | Parietal lobe | Glioma | Astrocytoma Grade IV | + + | None | Died | 6.175 |
| 17 | F | 30 | Frontal lobe | Glioma | Oligodendroglioma | + | None | Yes | 9.33 |
| 18 | M | 58 | Parietal lobe | Glioma | Glioblastoma multiforme | + + | Steroids | Yes | 2.49 |

[a]Any one (+) or more (+ +) of: nuclear pleomorphism; cellular immaturity; tumour angiogenesis; tumour necrosis. [b]To some extent recurrence depends upon lengths of clinical follow up or survival. [c]Kernohan grading I–IV (IV = highly malignant) *Proc. Staff Meet. Mayo Clin.*, **24,** 71–75.

**Table III** Control and unclassifiable samples

| Patient no. | Sex | Age | Site of specimen | Nature of specimen | Histological features | Signs of active growth | Drug treatment | Mean ESAF content μg collagen degraded $h^{-1}$ $mg^{-1}$ protein |
|---|---|---|---|---|---|---|---|---|
| 19 | F | 24 | Corpus callosum | White matter adjacent to arteriovenous malformation | Normal white matter | – | None | 1.12 |
| 20 | F | 39 | Lumbar spine | Tumour of spinal cord | Lipoma | – | None | 0.51 |
| 21 | F | 47 | Frontal lobe | Cortex from patient with brain haemorrhage | Normal cortex | – | None | 0.55 |
| 22 | M | 35 | Temporal lobe | Cortex from patient with head injury | Contused but 'normal' cortex | – | None | 0.30 |
| 23 | M | 55 | Cortex | | Normal cortex | – | None | 0.31 |
| 24 | M | 62 | Cortex | Cortex obtained post mortem — | Normal cortex | – | None | 0.04 |
| 25 | F | 66 | Cortex | patients died from myocardial infarction | Normal cortex | – | None | 0.1 |
| 26 | F | 77 | Cortex | | Normal cortex | – | None | 0.09 |
| 27 | F | 82 | Cortex | | Normal cortex | – | None | 0.04 |

umn and the peak eluting before the procollagenase peak was collected. This peak contained collagenase inhibited by the tissue inhibitor of metalloproteinase (TIMP). This complex has been shown to be activated by ESAF (McLaughlin et al., 1991). 50 $\mu$g $^3$H-labelled collagen was dissolved in 250 $\mu$l of 50 mM tris-HCl buffer (pH 7.6) containing 0.2 M NaCl and 0.01 M $CaCl_2$ and preincubated for 30 min at 36.5°C. During this time the collagen gelled. ESAF and latent collagenase in the above buffer were then added to give a final volume of 500 $\mu$l and the tube was incubated for 4 h at 36.5°C. During this time activation by ESAF of the latent collagenase to form an active enzyme occurred, leading to the degradation of $^3$H-labelled collagen and the release of radioactivity into the buffer. 6 M NaCl in 50 mM tris-HCl buffer was then added (250 $\mu$l) to precipitate any undegraded collagen which had been solubulised during the incubation. After incubation for a further 30 min, samples were centrifuged at 1700 $g$ for 30 min and the radioactivity present in the supernatant was determined by scintillation counting. Activation was linear up to the degradation of 30 $\mu$g collagen. Samples giving results higher than degradation of 25 $\mu$g collagen were diluted and rerun.

### Results

All tumours and control brain samples contained detectable quantities of a diffusible, low molecular mass, latent collagenase activating factor. Gel filtration chromatography of representative samples on Bio-Gel P-2, showed the active material to have an apparent molecular mass of 400.

When semi-purified fractions of the biopsy samples were tested on the chick yolk sac membrane, positive results were obtained. This indicates the presence of an angiogenic factor (Figure 1) and suggests the presence of ESAF in these samples.

The concentration of ESAF in control brain samples were of the same order as those present in bovine brain and retina and rat brain, but were considerably less than those found in human or bovine pineal glands (not shown). This suggests that there is no apparent species difference between bovine and human tissue in regard to levels of ESAF. Similar results have also been obtained from rats (not shown). ESAF levels present in brain tumours did not relate to the gender of the patients. However, in control samples there is an indication that there may be a correlation with low values and age. Analysis of results from tumours for which information on their growth characteristics was available indicated that neoplasms which were not actively growing had amounts of ESAF not significantly different from those in control brain (Figure 2). In contrast tumours showing morphological features of active growth had concentrations of ESAF considerably in excess of those found in control brain or in non-active tumours ($P = 0.01$ and 0.025 respectively) tumours (Figure 2). Thus in actively growing or malignant tumours where neovascularisation would be a pre-requisite for sustained growth ESAF levels were very significantly raised. Groups of tumours conventionally regarded as malignant (gliomas and astrocytomas) had ESAF levels considerably greater than the control brain samples ($P = 0.03$) or brain tumours usually considered to be benign such as pituitary tumours, meningiomas and acoustic neuromas (Figure 2). Analysis of tumours according to individual type indicated that only gliomas contained significantly more ESAF than control brain samples (Figure 3 and Tables I–III). Although meningiomas and pituitary tumours had mean values which were generally greater than control brains.

### Discussion

It is known that tumour tissue contains factors capable of inducing angiogenic activity (Shing et al., 1985; Folkman & Klagsbrun, 1987) and the production of many of these fac-

**Figure 1** Chick yolk sac membrane showing the response to **a**, a sample of ESAF derived from a glioma, **b**, an ESAF fraction isolated from a pituitary tumour and **c**, a control pellet containing no ESAF. Samples were applied in pellets consisting of methyl cellulose (4000 centapose: Sigma UK) and were photographed 24 h later.

**Figure 2** A comparison of ESAF concentrations in control human brain samples with those found in actively growing and non-actively growing tumours and with malignant and benign tumour types. The figures for malignant and benign tumours and active and non-actively growing tumours are averages of results in Tables I and II. Both malignant and benign tumours were significantly different from control brain ($P = 0.03$ and 0.04 respectively). Actively growing tumours were significantly higher than non-active tumours ($P = 0.025$). 1 unit of ESAF = the amount activating sufficient latent collagenase to degrade 1 μg collagen $h^{-1}$.

**Figure 3** A comparison of ESAF levels in control brain with acoustic neuromas, gliomas, meningiomas and pituitary tumours. Only the gliomas were significantly different from control brain (0.03). 1 unit of ESAF = the amount activating sufficient latent collagenase to degrade 1 μg collagen $h^{-1}$. A = Control; B = Acoustic Nerve; C = Glioma; D = Meningioma; E = Pituitary.

tors by tumour cells grown in culture has been confirmed (Folkman & Klagsbrun, 1987). Thus tumour extracts and conditioned medium from tumour cell cultures have been shown to exhibit growth-promoting activity in appropriate test systems (Folkman, 1977; Klagsbrun *et al.*, 1986). In some studies the mediators of angiogenic activity have been isolated and identified. Factors have been isolated which are closely related to fibroblast growth factors both in structure and activity (Shing *et al.*, 1985; Klagsbrun *et al.*, 1986). We have previously isolated tumour ESAF in our laboratories (Weiss *et al.*, 1979; Lye *et al.*, 1986). Fibroblast growth factors and ESAF have been extracted from normal adult tissue and from embryonic tissue. Other putative growth factors such as transforming growth factor alpha and gastrin releasing peptide appear only to be found in significant quantities in foetal and neoplastic tissues (Bothwick *et al.*, 1984; Caffey *et al.*, 1986; Wharton *et al.*, 1979). Few attempts have been made to compare the actual concentration of growth factors in normal brain tissue and with those in brain tumours although it has been shown that the angogenic activity of cerebrospinal fluid taken from patients with certain brain tumours is significantly raised compared with that in the cerebrospinal fluid from control patients (Pousa *et al.*, 1983).

In the present study we measured the concentrations of ESAF taken from 'control' (i.e. tumour free) brain tissue and compared it with that of ESAF in intracranial neoplasms (Figure 2). Control samples were obtained from patients with head injury when damaged brain was resected as part of their treatment or from autopsied patients. It is possible that angiogenic activity in brain tissue may be lost owing to biodegradation soon after death, but results obtained from the two control sources were reasonably comparable except that there appeared to be lower levels in older patients although this was not significant.

Although our numbers are small it appears that in general intracranial neoplasms have increased concentrations of ESAF compared with control samples. There is significant differences in ESAF levels when intracranial tumours regarded as 'actively growing' are compared to 'inactive' tumours ($P = 0.025$) (Figure 3). Those tumours considered to be clinically benign (e.g. acoustic neuromas, pituitary tumours, meningiomas) appear to have significantly lower concentrations of ESAF when compared with clinically malignant tumours (such as gliomas) (Figure 3) ($P = 0.03$). Some tumour samples obtained from the malignant group had ESAF levels comparable with those present in the pineal gland which has the highest level for normal tissues of the body (Taylor *et al.*, 1988). This is of interest as Korf *et al.* (1987) have identified in some brain neoplasms the presence of genetic markers which were previously thought to be confined to the pineal gland.

We are unable to comment as to whether there exists a correlation between ESAF concentration and patient survival for a given tumour type. This is because there are a large number of relevant factors we would have to consider, for example; age on diagnosis; specific histopathological characteristics; sex; treatment with steroid hormones; size and site of tumour at the time of biopsy and the extent of tumour removal. The relatively small number of patients and our short clinical follow-up also limits us in this respect.

It is probably significant, however that as well as those tumours generally regarded as having an unfavourable prognosis (e.g. glioblastoma multiforme) samples from certain 'benign' tumours had high ESAF levels. Certain meningiomas and pituitary tumours, though usually curable, may regrow and we were therefore not surprised that samples from some of these tumours had high ESAF levels. On the other hand, those tumours such as acoustic neuromas which almost never recur after total removal had ESAF levels close to the control values.

We conclude that intracranial neoplasms contain increased amounts of ESAF compared to normal brain tissue. Furthermore, the more actively growing tumours have higher concentrations of ESAF than the more slowly growing benign tumours. With further study it may be possible to use the concentration of ESAF in biopsy samples in order to give a more accurate prognosis for the outcome with respect to tumour type. It may also one day enable clinicians to devise therapeutic stratagems to influence tumour growth.

This work was funded by a grant from the North West Regional Health Authority, UK to R.H. Lye and J.B. Weiss. The authors would like to thank Dr Helen Reid, Lecturer in Neuropathology for histological examination of specimens and Dr Emir Benbow and Dr Paul Slater for the supply of autopsy samples.

### References

BOTHWICK, D.C., ROTH, K.A., EVANS, C.J., BARCHAS, J.D. & BENSCH, K.G. (1984). Gastrin releasing peptide, a mammalian analogue of bombesin, is present in human neuroendocrine lung tumours. *Am. J. Pathol.*, **117**, 195.

CAFFEY, R.J., SHIPLEY, G.D. & MOSES, H.L. (1986). Production of transforming growth factors by human colon cancer lines. *Cancer Res.*, **46**, 1164.

ELSTOW, S.F., SCHOR, A.M. & WEISS, J.B. (1985). Bovine retinal angiogenesis factor is a small molecule (Mr 600). *Invest. Ophthalmol. Vis. Sci.*, **26**, 74.

FOLKMAN, J. (1977). Tumour angiogenesis factor. *Sciences*, **23**, 442.

FOLKMAN, J. (1984). In *Biology of Endothelial Cells*. Jaffe, E.A. (ed.) p. 142. Nijhoff: Boston.

FOLKMAN, J. & KLAGSBRUN, M. (1987). Angiogenic factors. *Sciences*, **235**, 442.

GISSLOW, M.T. & MCBRIDE, B.C. (1975). A rapid sensitive collagenase assay. *Anal. Biochems.*, **68**, 70.

KLAGSBRUN, M., SASSE, J., SULLIVAN, R. & SMITH, J.A. (1985). Human tumor cells synthesise an endothelial cell growth factor that is structurally related to basic fibroblast growth factor. *Proc. Natl Acad. Sci. USA*, **83**, 2448.

KORF, H.-W., CZERWIONKA, M., REINER, J. & 4 others (1987). Immunocytochemical evidence of molecular photoreceptor markers in cerebellar medulloblastomas. *Cancer*, **60**, 1763.

LOWRY, O.H., ROSENBROUGH, M.J., FARRI, A.L. & RANDALL, R.J. (1951). Protein measurement with a Foilin phenol reagent. *J. Biol. Chem.*, **193**, 265.

LYE, R.H., ELSTOW, S.F. & WEISS, J.B. (1986). Neovascularisation of intracranial tumours. In *Biology of Brain Tumours*, Walker, J.D. & Thomas, D.G.T. (eds). p. 61. Martinus Nijhoff: Boston.

ODEDRA, R. & WEISS, J.B. (1987). A synergistic effect on microvessel endothelial cell proliferation between basic fibroblast growth factor and ESAF. *Biochem. Biophys. Res. Commun.*, **143**, 947.

POUSA, S.L., PASUCHI, J.M.V., FERRER, I., DOMENECH, J.M., POUSA, A.L. & ARRIBAS, F.R. (1983). Angiogenic activity in fluid samples from tumoural patients. *Cancer*, **52**, 1365.

SCHOR, A.M., SCHOR, S., WEISS, J.B., BROWN, R.A., KUMAR, S. & PHILLIPS, P. (1980). Stimulation by a low molecular weight angiogenic factor of capillary endothelial cells in culture. *Br. J. Cancer*, **41**, 790.

SHING, Y., FOLKMAN, J., HAUDENSCHILD, C., LUND, D., CRUM, R. & KLAGSBRUN, M. (1985). Angiogenesis is stimulated by a tumour derived endothelial cell growth factor. *J. Cell. Biochem.*, **29**, 275.

TAYLOR, C.M. & WEISS, J.B. (1984). The chick vitelline membrane as a test system for angiogenesis and antiangiogenesis. *Int. J. Microcirc. Clin. Exp.*, **3**, 337.

TAYLOR, C.M., MCLAUGHLIN, B., WEISS, J.B. & SMITH, I. (1988). Bovine and human pineal glands contain substantial quantities of endothelial cell stimulating angiogenesis factor. *J. Neural Trans.*, **71**, 79.

VAFARDIS, J., MEATS, J.E., REID, H., LYE, R.H. & WEISS, J.B. (1987). Preliminary assessment of the correlation between clinipathological features of intracranial tumours and the amount of extractable angiogenic activity: high and low Mr factors. In *Brain oncology, diagnosis and therapy*. Chatel, M., Darcel, F. & Pecker, J. (eds), p. 149. Martinus Nijhoff: Dordrecht.

WEISS, J.B., BROWN, R.A., KUMAR, S. & PHILLIPS, P. (1979). An angiogenic factor isolated from tumours: a potent low molecular weight compound. *Br. J. Cancer*, **40**, 493.

WEISS, J.B., HILL, C.R., DAVIS, R.J., MCLAUGHLIN, B., SEDOWFIA, K.A. & BROWN, R.A. (1983). Activation of procollagenase by a low molecular weight angiogenesis factor. *Biosci. Rep.*, **3**, 171.

WHARTON, J., PALAK, J.M., BLOOM, S.R. & 4 others (1978). Bombesin like immunoreactivity in the lung. *Nature*, **273**, 769.


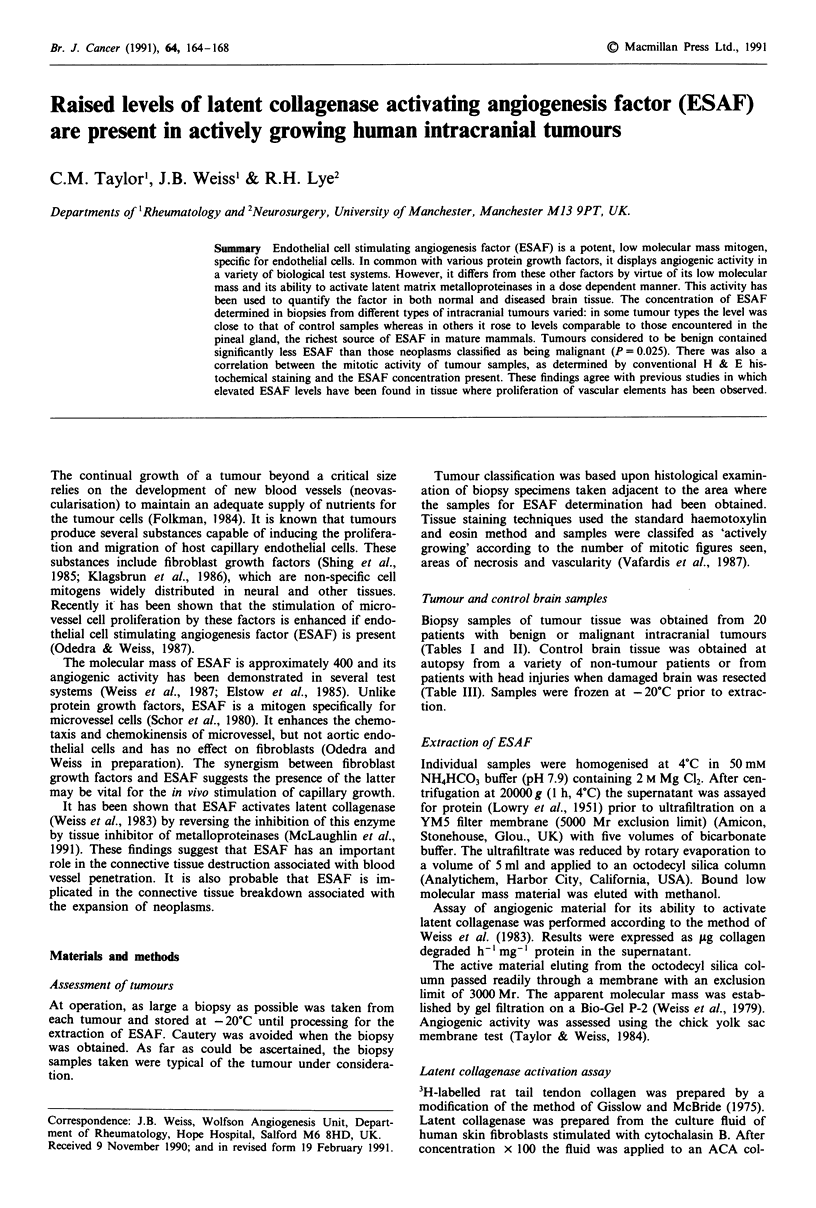

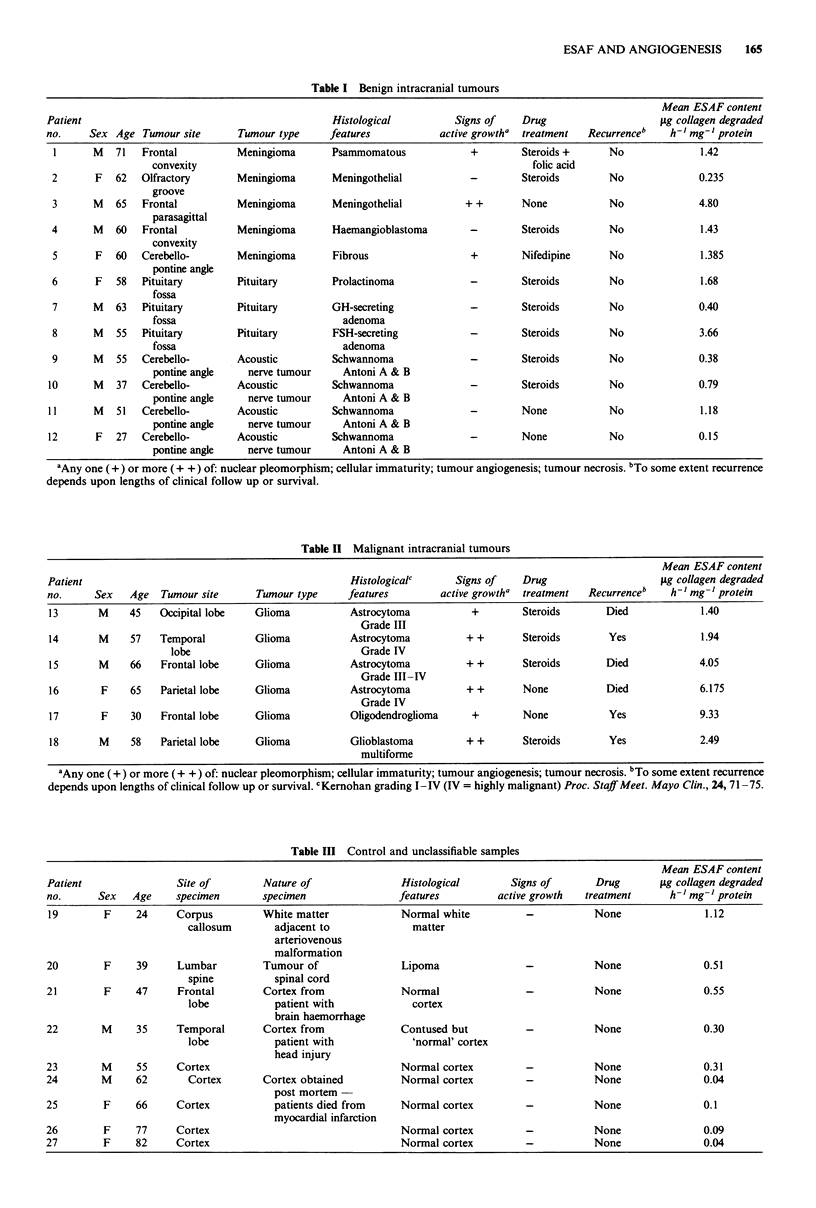

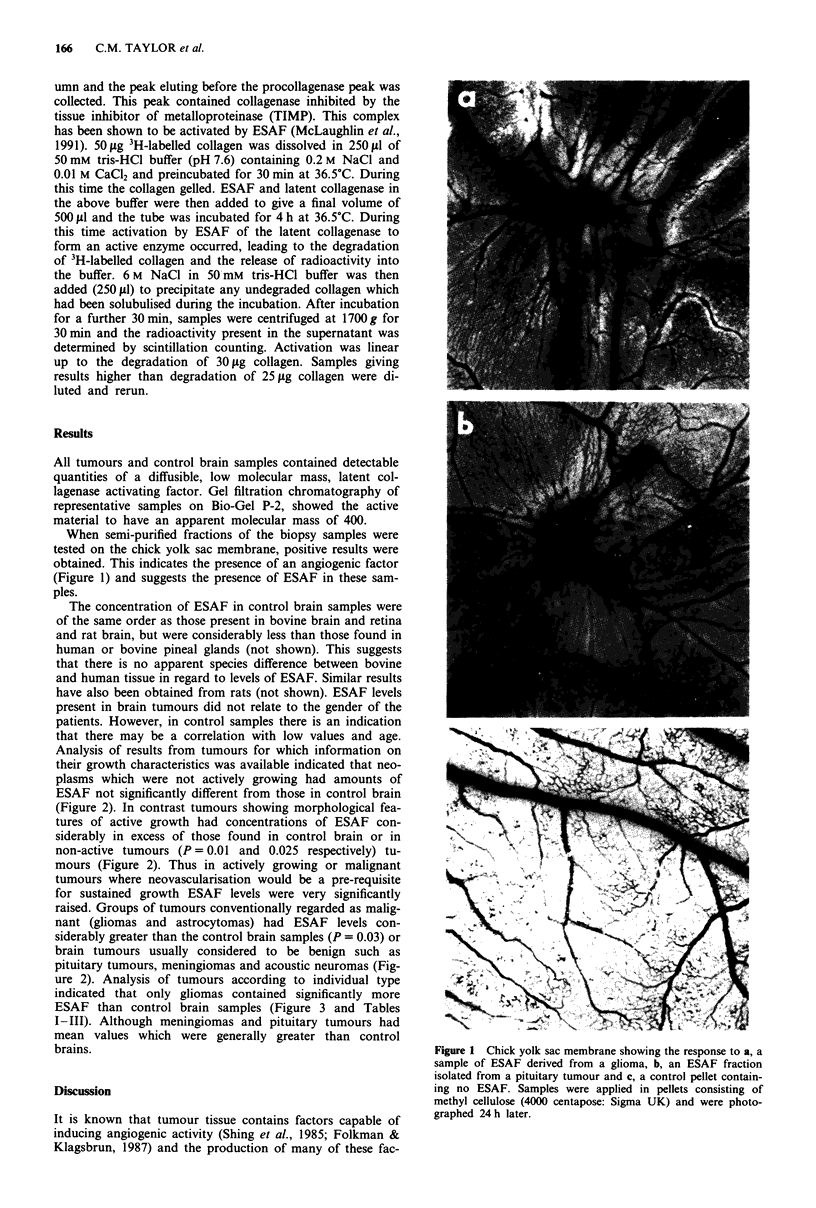

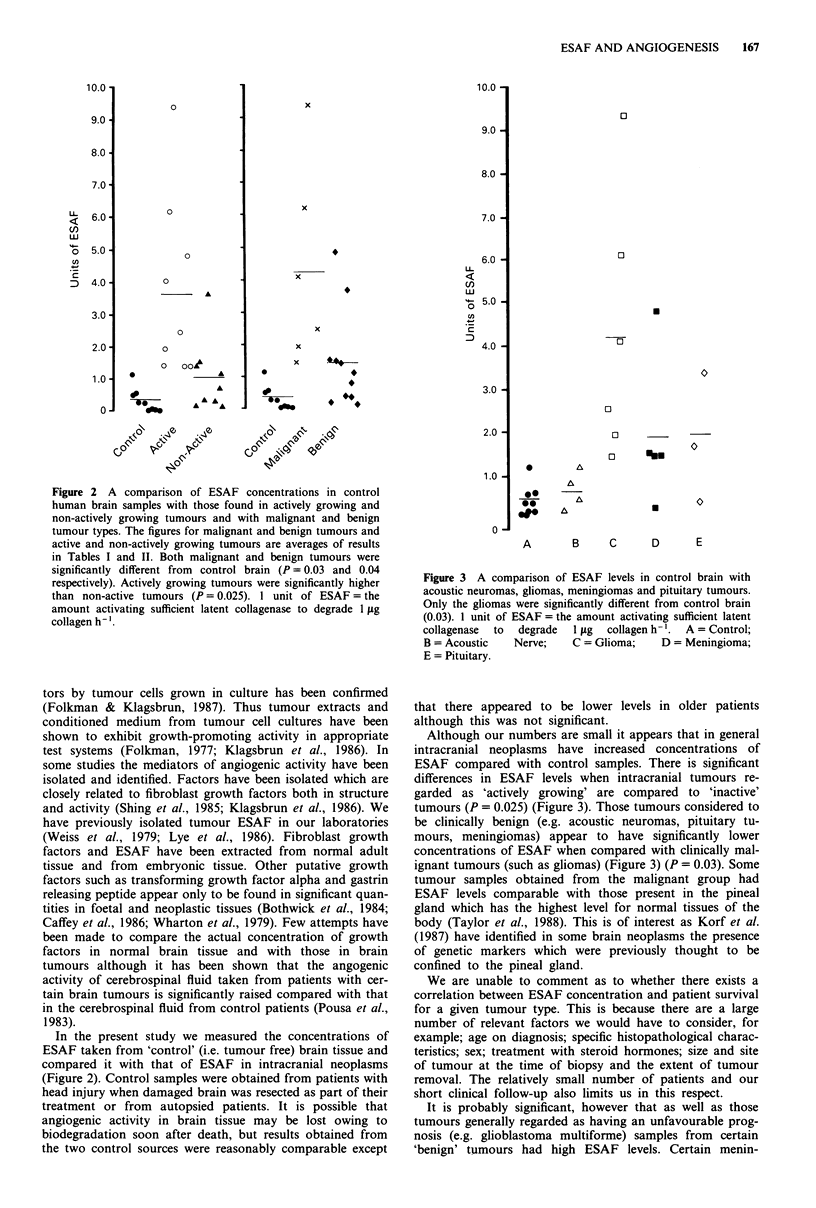

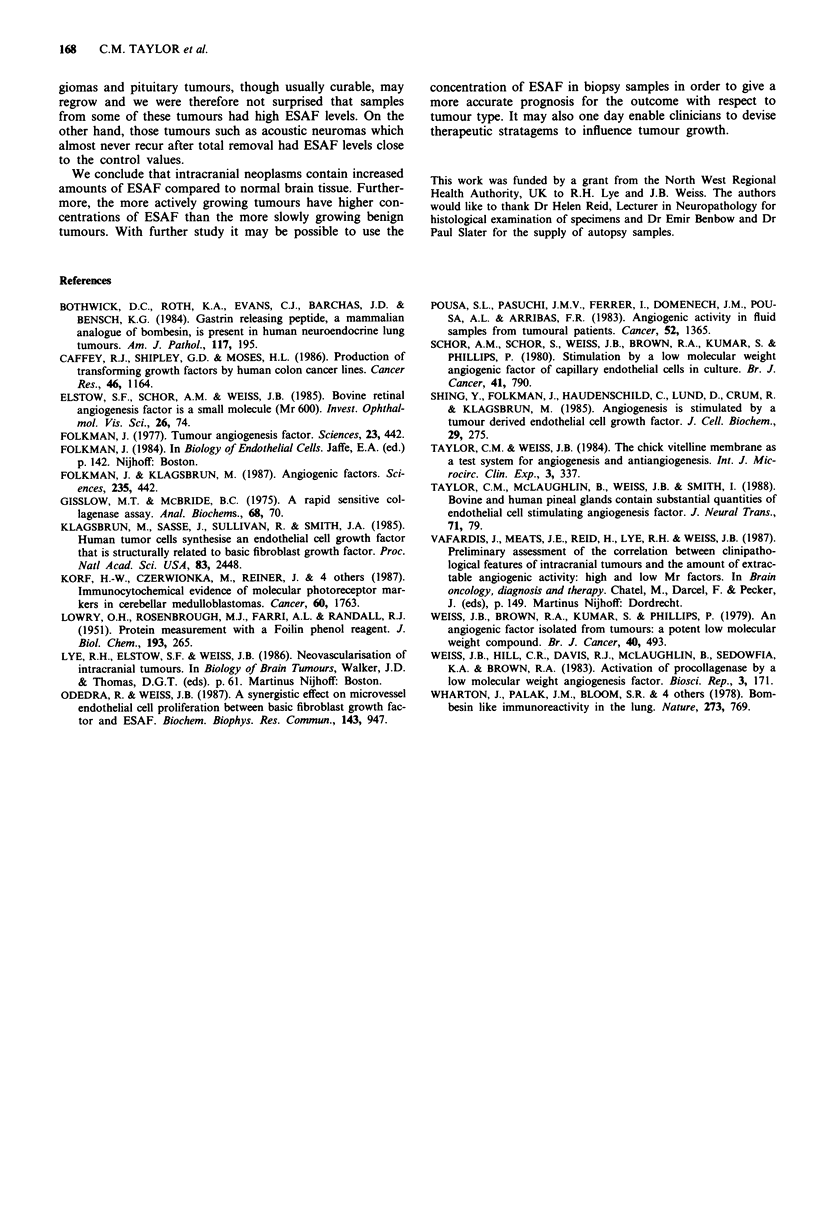


## References

[OCR_00702] Bostwick D. G., Roth K. A., Evans C. J., Barchas J. D., Bensch K. G. (1984). Gastrin-releasing peptide, a mammalian analog of bombesin, is present in human neuroendocrine lung tumors.. Am J Pathol.

[OCR_00708] Coffey R. J., Shipley G. D., Moses H. L. (1986). Production of transforming growth factors by human colon cancer lines.. Cancer Res.

[OCR_00713] Elstow S. F., Schor A. M., Weiss J. B. (1985). Bovine retinal angiogenesis factor is a small molecule (molecular mass less than 600).. Invest Ophthalmol Vis Sci.

[OCR_00723] Folkman J., Klagsbrun M. (1987). Angiogenic factors.. Science.

[OCR_00727] Gisslow M. T., McBride B. C. (1975). A rapid sensitive collagenase assay.. Anal Biochem.

[OCR_00731] Klagsbrun M., Sasse J., Sullivan R., Smith J. A. (1986). Human tumor cells synthesize an endothelial cell growth factor that is structurally related to basic fibroblast growth factor.. Proc Natl Acad Sci U S A.

[OCR_00737] Korf H. W., Czerwionka M., Reiner J., Schachenmayr W., Schalken J. J., de Grip W., Gery I. (1987). Immunocytochemical evidence of molecular photoreceptor markers in cerebellar medulloblastomas.. Cancer.

[OCR_00742] LOWRY O. H., ROSEBROUGH N. J., FARR A. L., RANDALL R. J. (1951). Protein measurement with the Folin phenol reagent.. J Biol Chem.

[OCR_00759] Lopez Pousa S., Vich i Pascuchi J. M., Ferrer I., Domenech J. M., Lopez Pousa A., Real Arribas F. (1983). Angiogenic activity in fluid samples from tumoral patients.. Cancer.

[OCR_00752] Odedra R., Weiss J. B. (1987). A synergistic effect on microvessel cell proliferation between basic fibroblast growth factor (FGFb) and endothelial cell stimulating angiogenesis factor (ESAF).. Biochem Biophys Res Commun.

[OCR_00762] Schor A. M., Schor S. L., Weiss J. B., Brown R. A., Kumar S., Phillips P. (1980). Stimulation by a low-molecular-weight angiogenic factor of capillary endothelial cells in culture.. Br J Cancer.

[OCR_00768] Shing Y., Folkman J., Haudenschild C., Lund D., Crum R., Klagsbrun M. (1985). Angiogenesis is stimulated by a tumor-derived endothelial cell growth factor.. J Cell Biochem.

[OCR_00779] Taylor C. M., McLaughlin B., Weiss J. B., Smith I. (1988). Bovine and human pineal glands contain substantial quantities of endothelial cell stimulating angiogenic factor.. J Neural Transm.

[OCR_00793] Weiss J. B., Brown R. A., Kumar S., Phillips P. (1979). An angiogenic factor isolated from tumours: a potent low-molecular-weight compound.. Br J Cancer.

[OCR_00798] Weiss J. B., Hill C. R., Davis R. J., McLaughlin B., Sedowofia K. A., Brown R. A. (1983). Activation of a procollagenase by low-molecular-weight angiogenesis factor.. Biosci Rep.

[OCR_00802] Wharton J., Polak J. M., Bloom S. R., Ghatei M. A., Solcia E., Brown M. R., Pearse A. G. (1978). Bombesin-like immunoreactivity in the lung.. Nature.

